# Engineering a More Thermostable Blue Light Photo Receptor *Bacillus subtilis* YtvA LOV Domain by a Computer Aided Rational Design Method

**DOI:** 10.1371/journal.pcbi.1003129

**Published:** 2013-07-04

**Authors:** Xiangfei Song, Yefei Wang, Zhiyu Shu, Jingbo Hong, Tong Li, Lishan Yao

**Affiliations:** 1 Shandong Provincial Key Laboratory of Energy Genetics, Qingdao Institute of Bioenergy and Bioprocess Technology, Chinese Academy of Sciences, Qingdao, China; 2 Key Laboratory of Biofuels, Qingdao Institute of Bioenergy and Bioprocess Technology, Chinese Academy of Sciences, Qingdao, China; Institut Pasteur, France

## Abstract

The ability to design thermostable proteins offers enormous potential for the development of novel protein bioreagents. In this work, a combined computational and experimental method was developed to increase the *T*_m_ of the flavin mononucleotide based fluorescent protein *Bacillus Subtilis* YtvA LOV domain by 31 Celsius, thus extending its applicability in thermophilic systems. Briefly, the method includes five steps, the single mutant computer screening to identify thermostable mutant candidates, the experimental evaluation to confirm the positive selections, the computational redesign around the thermostable mutation regions, the experimental reevaluation and finally the multiple mutations combination. The adopted method is simple and effective, can be applied to other important proteins where other methods have difficulties, and therefore provides a new tool to improve protein thermostability.

## Author Summary

The bacterial blue-light photoreceptor YtvA from *Bacillus subtilis*, which fluoresces in the presence or absence of oxygen, provides new opportunities to study oxygen-limited cellular systems. However its thermostability is poor, hindering its applicability in thermophilic systems. In this work, we develop an iterative combined computational and experimental method to significantly improve this protein's thermostability. The method is simple and effective, which in principle can be applied to other protein systems, thus adds a new tool to the protein engineering arsenal.

## Introduction

There is a considerable interest in proteins as therapeutics, biochemical reagents and catalysts. Many proteins tend to degrade on storage or use, and the instability hampers their applications in many aspects. Protein stability is very important to its application in biotechnology. For an industrial enzyme, higher thermostability means longer survival times and higher reaction temperature, which usually accelerates enzymatic catalysis and reduces contamination. Thus, proteins with good stability are highly desirable. Currently there are three main protein engineering methods, namely directed evolution [1], sequence consensus [2] and rational design [3]. Each of the three protein engineering methods has its own strength and weakness, and all have been used to improve protein thermostability with some success. Directed evolution uses methods such as DNA shuffling or error-prone PCR to create different mutations and employs quick screening essays to select the optimal ones. Sequence consensus involves the comparison of the targeted protein sequence to a series of homolog sequences and changes the amino acid at a specific position to that most frequently seen in the homologs. Rational design method relies on the 3D structure of a protein. Once the protein structure is known, the common rational design approaches include enhancement of core packing [4], removal of buried polar side chains [5], [6], mutation of charged surface residues [7]–[9], and introduction of new disulfide bonds [10]. However it is difficult to predict how well each approach works for a specific protein and the stability increase is likely caused by a combination of these effects. The success of rational design depends on the accurate understanding of the relationship between protein structure and stability. In principle, each residue in a protein can be substituted by nineteen natural amino acids but predicting the substitutions' effect on stability is very challenging. Many computational screening methods have been developed to pursue this goal, such as CC/PBSA [11], EGAD [12], FoldX [13], I-mutant 2.0 [14], Rosetta [15]–[17] and etc. Independent assessments of these methods suggested that all have high efficiency but only moderate accuracy [18]. A more recent study by Seeliger et. al. [19] using an alchemical free energy method to study point mutation effects on the thermostability of a barnase demonstrated much better accuracy. The free energy method utilizes a well-defined structure (e.g. from X-ray crystallography) to model the mutational effect on the folded state and a small peptide (e.g. GXG) to model the effect on the unfolded state. The thermodynamic cycle used in Seeliger's study provides a rigorous way to calculate the free energy change caused by a point mutation, though the method is computationally much more expensive than the quick screening methods.

Green fluorescent protein from the jellyfish *Aequorea Victoria* [20] and its derivatives are widely used in cell imaging to study gene regulation, protein syntheses, and other biochemical and cellular processes. However, in the catalytic formation of the chromophores, the requirement for molecular oxygen as a cofactor [21] hinders their applications for anaerobic microorganisms and oxygen-limited cellular microenvironments [22], [23]. The discovery of bacterial blue-light photoreceptor YtvA from *Bacillus subtilis* [24] and the SB2 protein from *Pseudomonas putida* [25], which fluoresce in the presence or absence of oxygen, provides new opportunities to study these oxygen-limited systems. YtvA is composed of 261 residues, with an N-terminal segment (residue 1–24), a LOV (light, oxygen, voltage) domain (25–126), and a C-terminal STAS (sulfate transporter/anti-σ factor antagonist, 148–258) domain. The LOV and STAS domains are connected by a long linker (127–147). The crystal structure of the LOV domain complexed with the chromophore flavin mononucleotide (FMN) has been determined for both the light and dark states [26]. The LOV domain forms a stable dimer *in vitro* [26], with a dissociation constant less than 10^−7^ M. Blue light absorption causes formation of a covalent bond between Cys62 and C(4a) of FMN. Drepper et al. mutated Cys62 to an alanine residue, which increases the fluorescence of *E. coli* cells by tenfold compared to the cells expressing the wild-type YtvA LOV domain [22]. However the engineered YtvA LOV (named FbFP hereafter, Flavin mononucleotide based Fluorescent Protein) only has moderate thermostability (see results below). A more thermostable FbFP would be desired for studying anaerobic microorganisms, especially thermophillic ones.

In this work we combined the quick method FoldX [13] and the slow but more accurate achemical free energy method to search for more thermostable FbFP mutants. Eighteen single mutants were selected for experimental testing, thirteen of which showed higher melting temperature 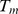. Subsequently, different single mutations were combined to yield even higher 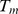 values.

## Materials and Methods

### FoldX prediction

The crystal structure of FbFP (residue 21–147 in subunit 1 and 21–146 in subunit 2, pdb code: 2PR5) [26] was used as the template for mutation, and the FoldX program was utilized to estimate mutational effects on protein thermostability. Firstly, the ligand FMN was removed and the structure was minimized using the ‘RepairPDB’ command. Then, single mutations were screened by substituting each residue with the other nineteen possible natural amino acids using ‘PositionScan’ command. Since the screening was performed separately for the two subunits, the predicted ΔΔG values for the same mutation by FoldX, slightly different due to the structure asymmetry, were averaged. The predicted stabilizing single mutations were built in both subunits using ‘BuildModel’ command for further free energy calculations.

### Free energy calculation

Molecular dynamics (MD) simulations were carried out using Gromacs 4.5 [27], [28], with the Gromos53A6 force field [29] and SPC/E water [30]. The starting FbFP structures were from x-ray crystallography (WT) or the FoldX calculations (mutants). All residues were assumed to be in their standard ionization states at pH 7.0. The proteins were solvated by adding 10.0 Å SPC/E water in a rhombic dodecahedron box and counter ions were used to neutralize the systems. Before free energy calculations, 1000 steps energy minimizations followed by 1 ns MD simulations at constant pressure (1 atm) and temperature (300 K) were performed to equilibrate the systems. The pressure was regulated using the extended ensemble Parrinello-Rahman approach [31], [32] and the temperature was controlled by a modified Berendson thermostat [33]. The Particle-Mesh-Ewald Method [34], [35] was used to evaluate the contributions of the long-range electrostatic interactions. A nonbonded pair list cutoff of 10.0 Å was used and the nonbonded pair list was updated every 5 steps. All bonds to hydrogen atoms in proteins were constrained by using the LINC [36] algorithm whereas bonds and angles of water molecules were constrained by SETTLE [37] algorithm, allowing a time step of 0.002 ps.

The folding free energy difference between two mutants X and Y was calculated from the difference of the free energies between the folded and unfolded simulations. Instead of directly calculating the folding free energies ΔG_1_ and ΔG_2_ for mutants X and Y, a thermodynamic cycle was built (Figure S2A) so that ΔG_3_ and ΔG_4_, corresponding to the free energy changes of mutating X to Y in unfolded and folded states, were computed through an alchemical process. Then the free energy difference ΔΔG defined as ΔG_2_−ΔG_1_ can be obtained by ΔG_4_−ΔG_3_, with a negative value indicating mutant Y is more stable than X and a positive value meaning X is more stable. It has been shown that for large mutations, direct transformation from X to Y (e.g. W→R) is challenging [19] and it becomes difficult to extract reliable free energy values. In this work, an intermediate state was built corresponding to a pseudo alanine residue (state I in Figure S2B), with the C_β_ atom type (zero charge) defined as CH1 instead of CH3 as in alanine in the Gromos53A6 force field. The transformation from X to Y was separated into two steps, X→I and Y→I, and the free energy difference of the two steps is the X→Y transformation free energy. The X (or Y)→I free energy change was calculated by the computational alchemy method [38]–[41], which is briefly described here. Taking X→I alchemy as an example, a λ dependent Hamiltonian H(λ) was introduced, with λ ranging from 0 to 1, H(0) = H(X) and H(1) = H(I). Since the only difference between the X state and the I state is the extra side chain atoms of residue X (except for alanine which has the same number of atoms as the I state), the computational alchemy corresponds to the annihilation of these atoms up to Cβ. A path was built to remove the side chain atom charges first followed by the van der Waals parameters elimination. The side chain charges were removed using linear λ transformation with 21 windows and λ set at 0.05*n* (*n* = 0, 1, …20). Then the van der Waals (VDW) interaction was eliminated using a soft core Lennard-Jones potential which converts the side chain atom types to dummy atoms [42], with α = 0.5, σ = 0.3, and a soft-core power of 1. These dummy atoms have the VDW radius and the depth of VDW potential well, as well as bonded parameter equal to zero. As a result, in the VDW transformation the bonded interactions with the side chain atoms (up to Cβ) of residue X were also removed. Forty-one windows were used for the VDW transformation, with λ equal to 0.025*n* (*n* = 0, 1, …40). Each window was run for 250 ps, and the last 200 ps data were used for free energy evaluation which was done using Bennett's acceptance ratio method [43]. Since X was mutated to I simultaneously in both subunits, the computed electrostatic and VDW free energies were scaled by 0.5. The total free energy change of the X→I alchemical transformation ΔG_(X→I)_ = ΔG_ele(X→I)_+ΔG_vdw(X→I)_, where ΔG_ele(X→I)_, ΔG_vdw(X→I)_ are free energy changes corresponding to Coulomb switching and VDW switching respectively. The same relationship applies for the Y→I transformation. The free energy change of the X→Y transformation, ΔG_4_ = ΔG_ele(X→Y)_+ΔG_vdw(X→Y)_ = (ΔG_ele(X→I)_−ΔG_ele(Y→I)_)+(ΔG_vdw(X→I)_−ΔG_vdw(Y→I)_). The error for each term was estimated as follows. We divided the 200 ps simulation data to five blocks and calculated the standard deviation of corresponding ΔG values (the free energy difference between two neighboring windows). We used the standard deviation as the estimation for the error of ΔG. The typical average correlation time for ΔH (the Hamiltonian difference between two neighboring windows) is about 3 ps (Figure S4), comparable to the value from the literature [44], suggesting the data from five blocks are independent in the simulation time span. However, longer correlation time that is not sampled well in the simulation may still contribute to the error of ΔG. When comparing the computed ΔG with the experimental value, one has to bear in mind that this error can be a source of the discrepancy.

Unlike the folded state, which has a well-defined structure, the unfolded state is difficult to model. It has been proposed to use a GXG tripeptide to mimic the unfolded state where X is the residue intended for mutation. This simple model yields reasonable results compared to the experimental ΔΔG values [19] and thus was adopted in this work. Similar to the folded state, the two step transformations were used to calculate the free energies and the same MD simulation protocol was employed for the GXG model. The free energy difference ΔG_3_ between the unfolded X and Y mutants can be calculated with the equation similar to that for ΔG_4_ as described above. The folding free energy change ΔΔG_(X→Y)_ = ΔG_4_−ΔG_3_ = ΔΔG_ele(X→Y)_+ΔΔG_vdw(X→Y)_ = (ΔG_ele(X→Y, F)_−ΔG_ele(X→Y, U)_)+(ΔG_vdw(X→Y, F)_−ΔG_vdw(X→Y, U)_), where subscript F (U) stands for the folded (unfolded) state. ΔΔG_ele(X→Y)_ and ΔΔG_vdw(X→Y)_ are the electrostatics and VDW contributions to the folding free energy differences between X and Y mutants. The error of ΔΔG_(X→Y)_ is propagated from that of ΔG. It is worth noting that ΔΔG_ele(X→Y)_ and ΔΔG_vdw(X→Y)_ depend on the path selected for the free energy integration thus are not uniquely defined. But the free energy decomposition provides valuable information which cannot be obtained by ΔΔG_(X→Y)_ alone [45]–[47].

### Sequence alignment analysis

*Bacillus Subtilis* FbFP was used as the initial target for sequence search in NCBI nonredundant protein database. Sequences were aligned using BLAST [48] and those with identity higher than 90% were excluded. A total of 83 sequences were collected and all have identity higher than 45% with *Bacillus Subtilis* FbFP. The frequency of one amino acid type at a specific site was defined as the total number of the occurrence of the amino acid at this site divided by 83, the total number of sequences.

### Cloning, expression and purification

The DNA encoding residues 20–147 of FbFP and a His tag at the C-terminus was ligated with the vector pET-30a digested with the same restriction enzymes. The ligation mixture was transformed into an *E. coli* strain DH10B. The correct coding sequence of the cloned *fbfp* gene was verified by DNA sequencing. The expression construct (pET-30a-*fbfp*) was then transformed into *E. coli* strain BL21 (DE3). All the mutations were made by PCR-based site-directed mutagenesis and verified by DNA sequencing. All the mutants were expressed and purified in a similar way. Briefly, 250 mL of LB medium containing 50 µg/mL kanamycin was inoculated with a fresh colony of expression strain BL21 (DE3) containing pET-30a-*fbfp*. The culture was grown at 37°C with vigorous shaking (∼200 rpm). When the OD_600_ of the culture reached 0.8, the expression of YtvA LOV was induced by the addition of IPTG to a final concentration of 0.5 mM. The culture was then grown for an additional 4 h. The cells were harvested by centrifugation, washed twice with water, resuspended in 20 mL of 5 mM imidazole, 0.5 M NaCl, and 20 mMTris-HCl buffer (pH 7.9), and disintegrated by three passes through a French press. The disintegrated cells were centrifuged (9600 g, 4°C, 20 min) and the resulting supernatants were purified by Ni-NTA affinity chromatography (Novagen) to homogeneity as determined by sodium dodecyl sulfate polyacrylamide gel electrophoresis (SDS-PAGE). Protein concentration was determined by Bradford protein assay [49].

### Fluorescence measurements and data analysis

The decrease of FMN fluorescence when heating FbFP to a higher temperature allows one to monitor protein folding and unfolding by fluorescence spectroscopy (Figure S3). By setting the excitation wavelength at 450 nm (maximum absorption frequency at room temperature [24]), the maximum change of fluorescence on thermal denaturation was obtained with an emission wavelength of 506 nm (Figure S3). The final protein concentration was 4.2 µM of monomer. Spectroscopic measurements were carried out in a thermostated cuvette holder and the temperature was gradually increased. For each data point at different temperature, a delay of 10 min was applied before performing fluorescence measurement to allow the full equilibration between folded and unfolded states. The errors were estimated based on duplicated measurements. The thermal denaturation of FbFP is reversible based on the observation that heating the protein to a higher temperature (e.g. 70°C) attenuates the fluorescence by more than 80%, but it is fully recovered when the protein is cooled to room temperature. The Gibbs free energy of unfolding at a specific temperature 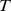 is written as [50],
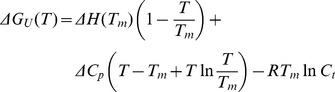
(1)where 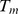 is the melting temperature (half of the protein population is unfolded), Δ*H* is the unfolding enthalpy at 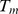. Δ*C_p_* is the difference of heat capacity in the folded and unfolded state which is assumed to be temperature independent. *C_t_* is the total protein monomer concentration. This equation shows that the Gibbs free energy as well as the melting temperature is related to the protein concentration [50]. Thus, in this work for different mutants, the same concentration (4.2 µM) was used to eliminate this effect. The fluorescence intensity (*I_F_*) at specific temperature can be expressed as [51],
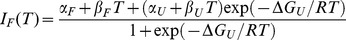
(2)where *α_F_* (*α_U_*) is the fluorescence of the folded (unfolded) state at zero K, and *β_F_* (*β_U_*) is the slope of the corresponding temperature dependence. Incorporating eq. 1 to 2 shows there are a total of seven parameters (Δ*H*, 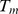, Δ*C_p_*, *α_F_*, *α_U_*, *β_F_*, *β_U_*) to be fitted to experimental fluorescence intensities. A simpler five-parameter model was also tested (Δ*H*, 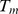, Δ*C_p_*, *α_F_*, *α_U_*, *β_F_* = *β_U_* = 0). Before data fitting, fluorescence intensities were scaled so that the maximum value was equal to 100. The fitting for FbFP using seven parameters yielded a reduced χ^2^ of 14.7 while fitting to the five-parameter model gave χ^2^ of 17.9, from which an F statistic value of 1.31 was obtained. This number was smaller than the critical *F*_(2,12)_ value of 3.88 (α = 0.05), indicating the complex seven-parameter model did not yield a significantly better fit. For all other mutants, the five-parameter model fittings also produced satisfactory results, and thus were selected for all fluorescence data analysis throughout this work. By adding random Gaussian distributed noise to the experimental data (with the standard deviation equal to the fluorescence measurement error), 100 synthetic data sets were created and fitted to estimate the errors of the fitting parameters. All the fittings were performed using an in-house script. The results show that Δ*C_p_* and Δ*H* have a considerable error derived from Gaussian noise (Table S1), but the error of Δ*G* is much smaller, with an average of 0.14 kcal/mol for all single mutants (Table 1). It is known that the Δ*C_p_* parameter is difficult to determine with the fitting of protein thermal denaturation fluorescence curve. Apparently the errors of Δ*H* and Δ*C_p_* cancel out each other. Thus, Δ*G* can be determined with accuracy higher than Δ*H* and Δ*C_p_*.

**Table 1:** Predicted and experimental ΔΔGs for different mutant proteins.

	ΔΔG(Coulomb switching)	ΔΔG(VDW switching)	ΔΔG(Total)	ΔΔG(Experimental)
H22K	−0.0	−0.6	−0.6±0.65	−0.3±0.03
H22W	−0.6	−2.2	−2.8±0.67	−1.2±0.06
V25I	0	−2.3	−2.3±0.22	−0.4±0.07
T30M	−1.8	−3.7	−5.5±0.63	−0.5±0.06
A33Y	−0.7	−1.5	−2.2±0.35	−0.5±0.05
T50M	−1.1	−0.7	−1.8±0.38	0.50±0.14
T54Y	1.4	−4.0	−2.6±0.43	−0.2±0.02
A81M	0.1	−1.3	−1.2±0.37	0.4±0.19
V88L	0	−1.1	−1.1±0.28	0.2±0.21
V90I	0	−1.3	−1.3±0.28	0.7±0.04
L106M	−3.9	0.8	−3.1±0.50	N/A^a^
N107F	−0.3	−2.0	−2.3±0.73	−0.9±0.07
N107Y	−0.4	−2.5	−2.9±0.70	−0.8±0.08
D109E	−7.1	−0.8	−7.9±1.17	−0.3±0.14
M111F	−1.8	−0.6	−2.4±0.64	−2.9±0.36
V120I	0	−1.5	−1.5±0.26	−1.4±0.60
N124F	−0.4	−3.2	−3.6±0.54	−1.1±0.03
N124Y	−1.2	−3.0	−4.2±0.41	−2.6±0.22

a ΔΔG was not determined due to the weak fluorescence of the sample at room temperature.

## Results/Discussion

### Computational screening

Starting from the crystal structure (pdb code: 2PR5), 2394 single mutants (126×19, saturation mutagenesis) were built and quickly screened by FoldX, which predicted 712 mutants from 94 sites have improved thermostability. Because it is very time consuming to do free energy calculations for all the mutants, the number of mutants was reduced to 103 using the following criteria: 1). For each site, if several mutations show improved thermostability, only the ones with relatively larger ΔΔG are selected. 2). For mutations involving charged residues, only those on surface that may form salt bridges are selected. Free energy calculations suggest that 70 of 103 mutants are more stable than the wild type (WT) FbFP, among which 40 mutants from 22 sites have ΔΔG more negative than −1 kcal/mol. The distribution of the 22 sites is shown in Figure S1.

### Experimental validation

**Figure 1:**
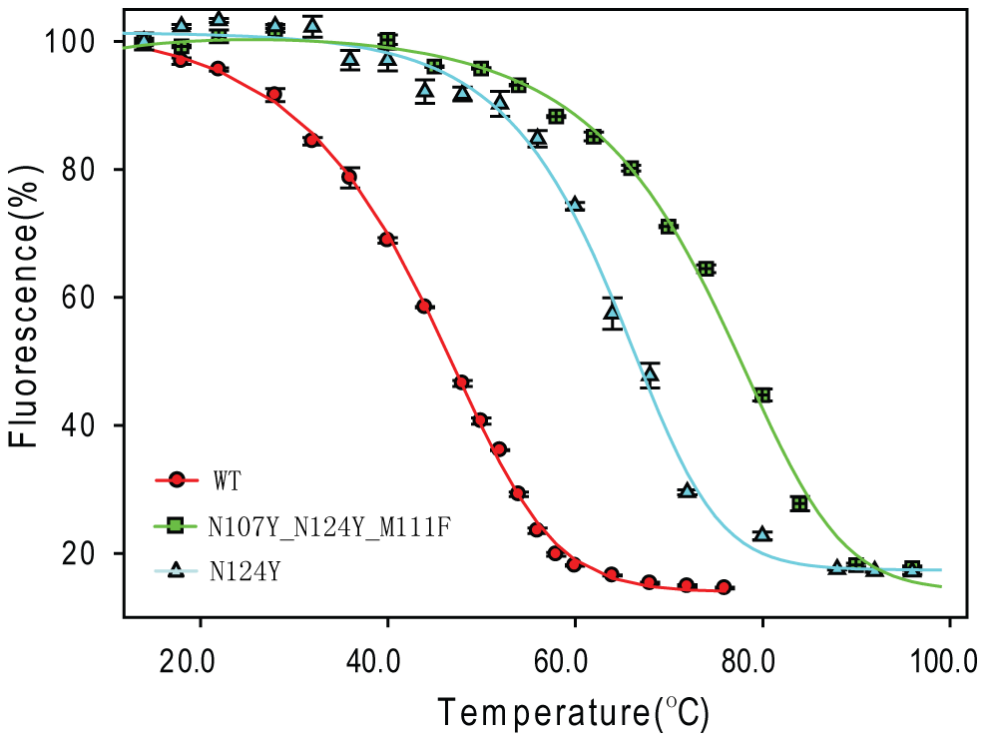
Thermal denaturation of the WT FbFP, the single point mutant N124Y, and the triple mutant N107Y-N124Y-M111F. The fluorescence intensity of the bound FMN is used to monitor the protein denaturation. As can be seen, the mutants have higher percentages of fluorescence at elevated temperature than WT suggesting mutations increase FbFP thermostability.

**Figure 2:**
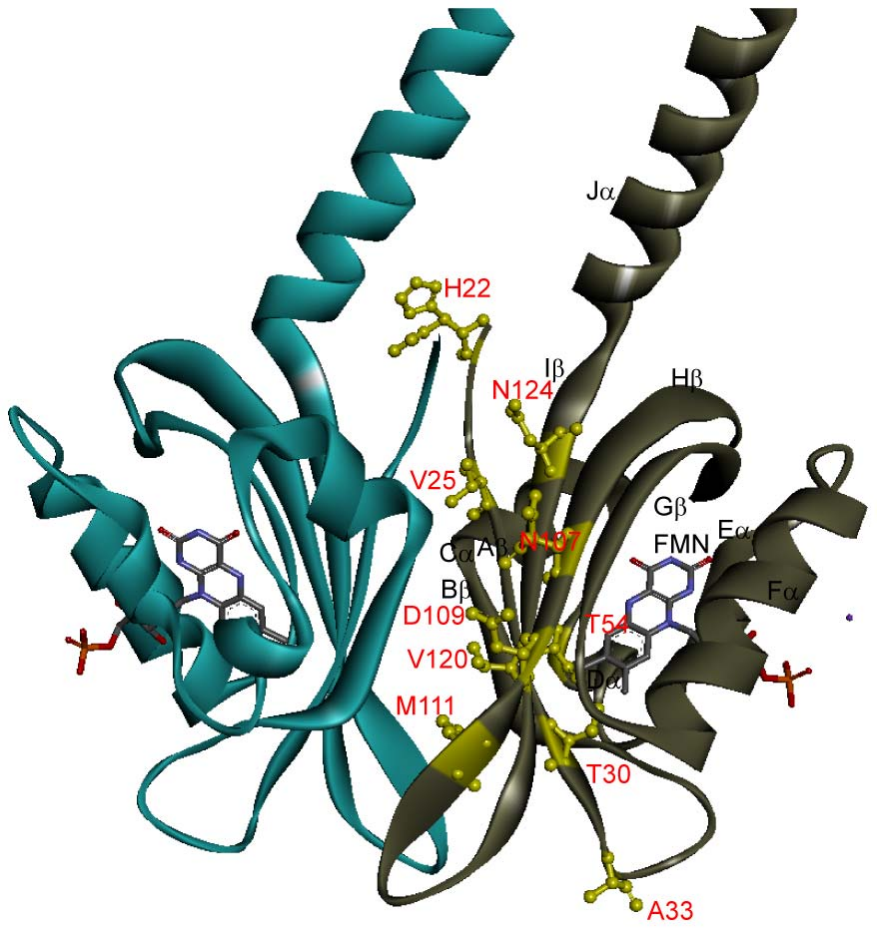
Locations of mutated sites exhibiting improved thermostability. WT residues of the mutated sites are highlighted in yellow and labeled in red. The two subunits are drawn in grey and dark cyan respectively. Residues H22, V25, N107, D109, M111, V120 and N124 are from the dimer interface. The figure was drawn based on FbFP x-ray structure 2PR5 by using Discovery Studio Visualizer program.

Thirteen mutants that have relatively more negative ΔΔG were tested experimentally, including V25I, T30M, A33Y, T50M, T54Y, A81M, V88L, V90I, L106M, N107F, D109E, V120I, and N124F. Melting temperatures of the wild-type (WT) and mutants were determined (see Materials and Methods) and are listed in Table 2. The WT FbFP has a 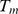 of 42.8°C, indicating a moderate thermostability. The temperature fluorescence plot suggests at 37°C, 25% of fluorescence is already lost compared to that of 14°C which is the starting temperature of measurements (Figure 1). Eight of the tested mutants displayed improved stability (Table 2), whereas four mutants are less stable and one mutant L106M loses fluorescence at room temperature, presumably because L106 points to the active site and the mutant might perturb the FMN binding pocket. The success rate of 62% is encouraging, highlighting effectiveness of the computational screening. Locations of the eight thermostable sites with higher 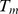 are shown in Figure 2. As can be seen, five sites are from the dimeric interface including V25, N107, D109, V120 and N124, suggesting that forging stronger interactions between subunits is an effective way to increase FbFP thermostability.

**Table 2:** Melting temperatures of WT and mutant FbFP.

FbFP	*T*_m_(Celsius)	FbFP	*T*_m_(Celsius)
WT	42.8±0.3	N107F	50.9±0.3
H22K	45.3±0.2	N107Y	52.0±0.5
H22W	49.4±0.2	D109E	46.8±2.0
V25I	46.6±0.5	M111F	56.5±1.0
T30M	47.3±0.4	V120I	54.2±0.9
A33Y	46.6±0.3	N124F	50.2±0.1
T50M	36.4±1.7	N124Y	63.8±0.1
T54Y	43.7±0.1	N107F-N124F	59.3±0.8
A81M	39.8±2.3	N107Y-N124Y	69.5±0.1
V88L	41.0±2.5	N107Y-V120I	56.4±0.6
V90I	33.6±1.1	N107Y-N124Y-H22W	67.5±0.2
L106M	N/A^a^	N107Y-N124Y-M111F	74.9±0.2

a *T*_m_ was not determined due to the weak fluorescence of the sample at room temperature.

**Figure 3:**
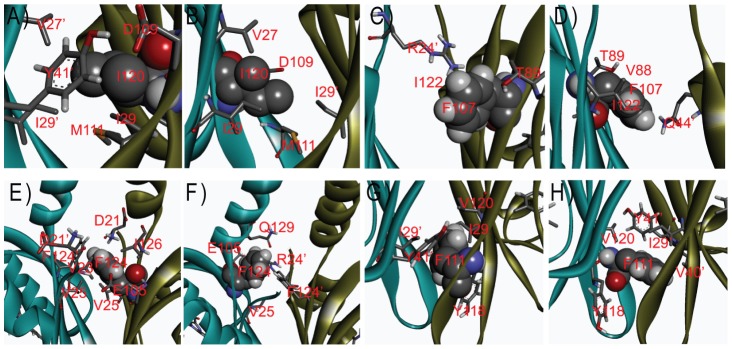
Residues in close contact with I120 (A, B), F107 (C, D), F124 (E, F) and F111 (G, H) are labeled where (A, C, E, G) are from subunit 1 and (B, D, F, H) are from subunit 2.

Among the tested mutants, V120I has the highest 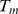 (54.2°C), 11.4°C higher than that of the WT. It is surprising that mutating a valine to an isoleucine, equivalent to substituting one H_β_ atom with a methyl group, has such a dramatic effect. In the free energy calculation, the force field GROMOS53A6 was used where the side chain charges are zero for these two residues. So the stabilization of the methyl substitution is exclusively from the van der Waals interaction (Table 1). The analysis of V120I MD trajectory suggests that in subunit one this methyl is in contact with V27′ and I29′ while in subunit two it contacts I29′, I29 and M111 (prime denotes residues from the neighboring subunit, Figure 3A and B, table S2). The contact differences in two subunits reflect the asymmetry of the dimer.

N107F and N124F have second (50.9°C) and third (50.2°C) highest 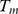 among the thirteen mutants. The free energy calculation suggests that the stabilization of the mutants is mainly from stronger van der Waals interaction (Table 1). It is worth noting that the free energy decomposition to the electrostatics and van der Waals is only an approximate process [46], [52], [53]. In this work, we decompose the free energy to two components, the free energy change of Coulomb switching and that of VDW switching, slightly different from the literature. Though the decomposition is exact, the values depend on the integration path and the process should only be considered as a qualitative analysis. However it can be linked to the structure and interaction more easily, thus provides important insight about the enthalpic part of the free energy. The MD simulation of the WT shows that N124 from subunit 1 is in contact with R24′ and V25, whereas N124 from subunit two is in contact with V23′, V25 and E105. In the mutant N124F, F124 forms much more contacts than WT N124 in both subunits (Figure 3C and D, Table S2). But for N107F, the difference is less dramatic. Free energy calculations indicate van der Waals contributes more than the electrostatics to ΔΔGs for all stabilizing mutants except D109E, revealing the importance of enhancing core packing to protein stability. The carboxyl group of D109 forms a hydrogen bond as an acceptor with the phenol group of Y141′. Mutating D109 to a glutamic acid extends the side chain and an extra hydrogen bond is formed with the N107 side chain amide (Table S3).

### Rational design of more mutants

The high success rate of the stable interface mutants prompts us to search for more candidates in the region. Most of the discovered stabilizing mutants including V25I, N107F, D109E, V120I and N124F are from the middle of the beta-strands that form the interface. In searching for other stabilizing mutants, more effort was put in interface regions away from the middle part because the combination of mutants close to each other may not be additive. Residues H22, M111 were identified. H22 was mutated to a lysine to promote a potential salt bridge formation with E105′ or E133′, or to a tryptophan to form more contacts. M111 was mutated to a phenylalanine to increase this residue's contacts with the neighboring subunit. Free energy calculations demonstrate that all three mutants have negative ΔΔG values, suggesting they are more stable than the WT. Inspection of the mutants N107F and N124F MD snapshots implies it is possible to further stabilize the protein by mutating F107 or F124 to a tyrosine to form more favorable electrostatic interactions with the surroundings. This observation is supported by the free energy calculations that N107Y (N124Y) has a more negative ΔΔG value than N107F (N124F) and the difference is mainly from electrostatic interactions (Table 1).

Five mutants H22K, H22W, M111F, N107Y and N124Y were tested experimentally and the corresponding 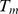 values were listed on Table 2. All five mutants were proved to be more stable, consistent with the computational results, among which N124Y has the highest 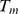 (63.8°C), 21.0°C higher than that of the WT. The analysis of the N124Y MD trajectory suggests besides close contacts with various residues (Table S2), N124Y forms extra hydrogen bonds. Specifically, in subunit one the side chain hydroxyl group of N124Y is h-bonded to V23′ and V25′, and in subunit two it is h-bonded to V23′ and Q129 (Table S3). These hydrogen bonds are expected to contribute the most to the 13.6°C 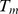 increase of the F to Y mutation. In comparison, N107Y is also more stable than N107F but the difference is much smaller, only ∼1°C. M111F has a 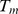 of 56.5°C, 13.7°C higher than the WT. MD simulation suggests F111 in subunit one forms close contacts with I29, I29′, Y41′, Y118 and V120, and in subunit two this residue is in contact with I29′, V40′, Y41′, Y118 and V120 (Figure 3G and H,table S2). H22K and H22W have 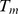 values 3°C and 7°C higher than the WT respectively. MD trajectory shows K22 forms salt bridges (Table S4), and W22 forms several contacts with the surroundings (Table S2).

### Multiple mutations combination

About a dozen mutants have 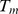 higher than that of the WT, providing an opportunity to improve the thermostability of the protein further by combining multiple mutations. But there are many ways of combining them. Two factors were considered, the Δ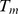 (melting temperature difference with the WT) and the distance between different mutants. Several multiple mutants were tested, among which double mutant N107Y-N124Y displays better thermostability than both N107Y and N124Y, and triple mutant N107Y-N124Y-M111F has its 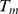 higher than all three single mutants (Table 2). However, not all the multiple mutants have higher thermostability than the single or double mutant. For example, N107Y-N124Y-H22W has a 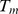 value 2°C lower than that of N107Y-N124Y, though H22W has a 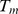 7°C higher than that of the WT. Similarly, N107Y mutation increases the 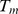 by 9°C, but N107Y-V120I is only marginally more stable than V120I (Table 2). So the additivity of the thermostability seemly holds true only for certain multiple mutants. N107Y-N124Y-M111F is the mutant with the highest 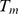 presented in this work, which is 31°C higher than that of the WT. To ensure that the triple mutations do not compromise the fluorescence brightness of the protein, its fluorescence quantum yield was determined, *Q*_F_ = 0.38, comparable to that of the WT FbFP 0.39 [22].

### Performance of the computational methods

**Figure 4:**
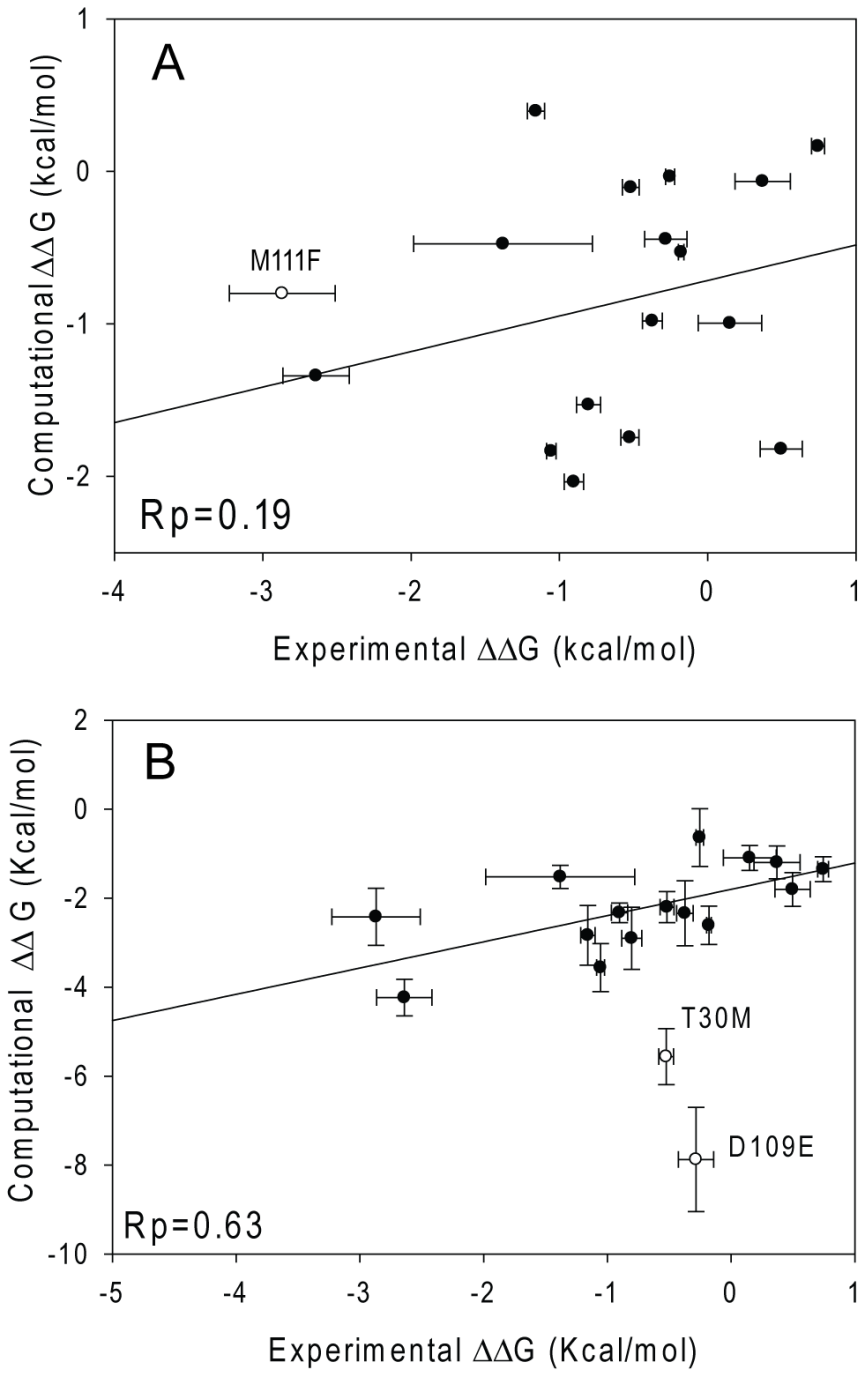
Correlation between the experimental and computational ΔΔG by using FoldX (panel A) and the free energy method (panel B). The best fit line is ΔΔG_com = 0.233ΔΔG_exp −0.72 kcal/mol in panel A, and ΔΔG_com = 1.38ΔΔG_exp −1.42 kcal/mol in panel B.

FoldX method provides a quick way to evaluate the mutational effect on the protein stability. It takes about 82 hours to screen the amino acids at each site for FbFP (∼2400 mutations) in a dual processor (2.9 GHz Intel) computer. In this work, FoldX predicts the thermostability of twelve out of seventeen mutants correctly, suggesting that overall FoldX is effective in identifying stable mutants. Three mutants, T50M, A81M and V88L, which are less stable than WT, were not predicted correctly. Furthermore, H22W and M111F were predicted less stable than the WT with the ΔΔG of 0.40 and 4.25 kcal/mol respectively but the experimental ΔΔGs are −1.16 and −2.87 kcal/mol. Further inspection suggests that steric clashes exist for the M111F mutant with its surroundings. By using the last MD snapshot of the 1 ns NPT M111F simulation as the FoldX input, a ΔΔG of −0.70 kcal/mol was obtained indicating M111F is more stable. For mutations causing steric clashes, it has been recommended to either soften the VDW repulsion [16] or relax the backbone atoms slightly [15] which is not allowed in FoldX. The correlation between the FoldX predicted and experimental ΔΔG is shown in Figure 4A. A Pearson correlation coefficient R_P_ of 0.19 was obtained, suggesting that FoldX only provides qualitative information of the relative thermostability. For mutations at the same site, e.g. N107Y/F or N124Y/F, FoldX also predicts the relative thermostability in the wrong trend. For example, N124Y is significantly more stable than N124F but FoldX predicts ΔΔG of −2.68 kcal/mol for the former and −3.67 kcal/mol for the later.

Free energy calculations, derived from statistical mechanics, offer a more rigorous way to estimate mutational effect on thermostability but with a much slower speed. A single point mutation of FbFP needs 440 processor (AMD 1.9 GHz) hours, about 13,000 times slower than the FoldX approach. Thirteen of seventeen mutants were predicted with the correct sign of ΔΔG, except T50M, A81M, V88L and V90I. The predicted and experimental ΔΔGs are shown in Figure 4B. Two mutants, T30M and D109E, are apparent outliers. Excluding these two, a correlation coefficient R_P_ of 0.68 was obtained with the best fitted line of ΔΔG (predicted) = 0.59ΔΔG (experimental) −1.80 kcal/mol. A negative intercept suggests that free energy calculations systematically overestimate the stability of the mutants, which may be caused by the force field error. Excluding the two outliers T30M and D109E, the mean absolute error is to 1.7 kcal/mol. Applying the linear correction reduces the error to 0.93 kcal/mol, comparable to results in the literature [19]. Compared to FoldX, the free energy method is more accurate and yields semiquantitative results. But there is still room for improvement.

There are a total of 13 stabilizing mutants from 10 sites (Table 2). It is interesting to see that 7 of the 10 sites are from the dimeric interface, except T30, A33 and T54. Unlike the interface, mutants around these three residues have not been thoroughly examined. In fact, about 2/3 of the 40 stabilizing mutants predicted by the free energy method (Figure S1) are not from the interface region among which about half of the mutation sites have not been tested experimentally. More effort will be spent to search for single mutants away from the dimeric interface and tune computational methods for screening multiple mutants.

### Consensus analysis

A multiple sequence alignment was carried out for a total of 83 FbFP sequences from the NCBI protein database. It is striking that none of the 13 stable mutants discovered in this work have the highest occurrence frequency (defined as the probability to have a specific amino acid at a particular site) at the mutated site (Table S5). For example, for residue 120, valine has the highest occurrence frequency of 78.3% which is much larger than that of isoleucine 15.7%, but V120I improves the thermostability by 11.4°C. In fact the majority of the stable mutants have the occurrence frequency at mutated sites smaller than that of the WT. This finding strongly suggests that FoldX and free energy calculations explore very different sequence spaces from the consensus analysis method.

### Conclusion

**Figure 5:**
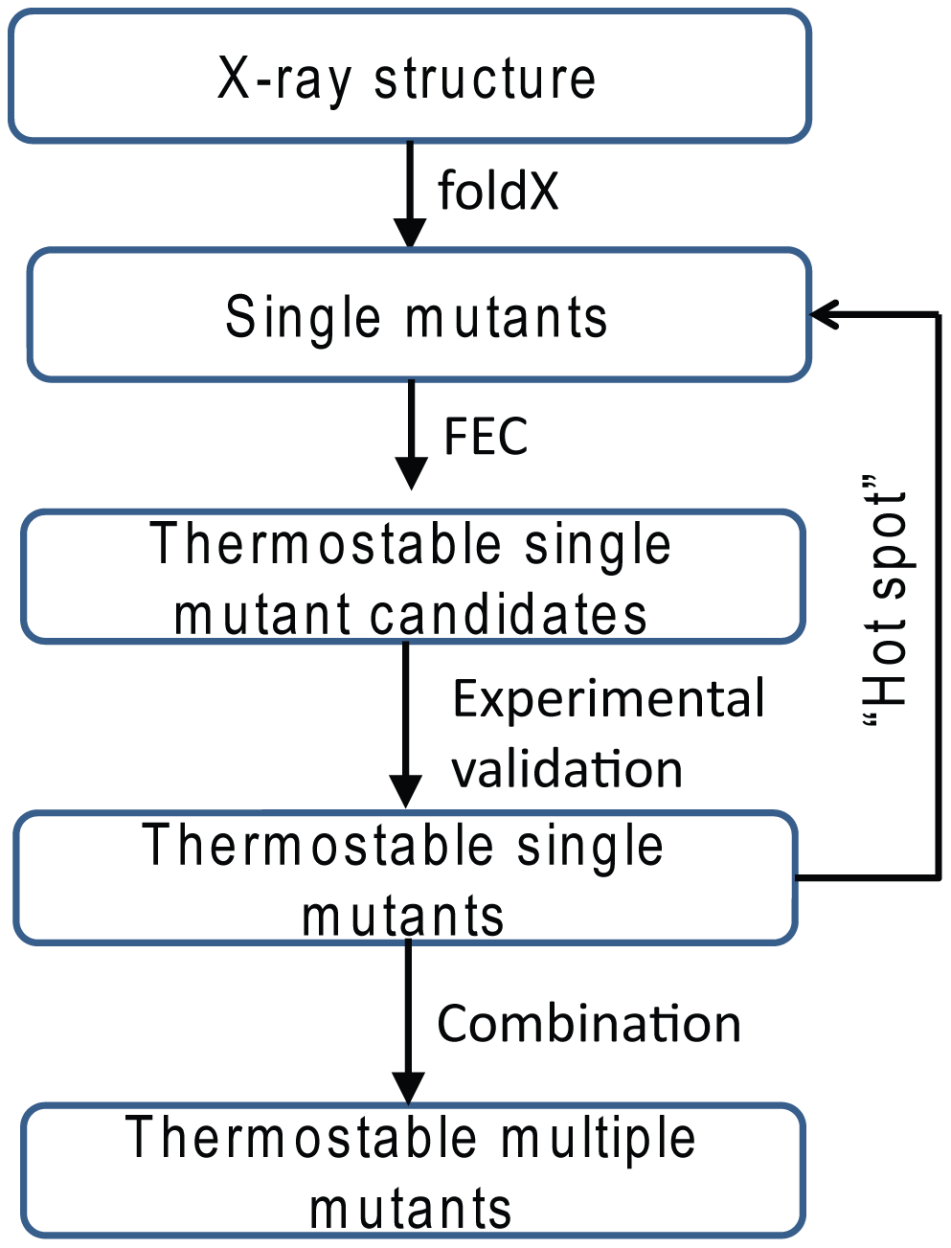
Flowchart of designing thermostable FbFP mutants. Briefly, FoldX followed by FEC (free energy calculation) are used to search for potential thermostable single mutants, from which a dozen are selected for experimental tests. The distribution of thermostable mutants is analyzed to identify the “hot spot”. Then more mutants in the “hot spot” are calculated by FEC and those predicted to be more stable are tested by experiments. Finally all stabilizing mutants are pooled together and multiple mutants are combined to further improve the protein's stability.

In this work, we developed a protocol to engineer a more thermostable FbFP. Firstly, FoldX followed by free energy calculations are performed to identify stable single mutant candidates. Secondly, mutants predicted with more negative ΔΔGs are selected for the experimental validation. For FbFP, the majority of stable mutants are from the dimeric interface which is a “hot spot” of the protein. Thirdly, more single mutants from the “hot spot” are selected and verified by free energy calculations first and those with more negative ΔΔGs are subject to experimental tests. Finally, stable single mutants are combined to further improve the protein thermostability. A flow chart of the protocol is shown in Figure 5. Thirteen out of eighteen tested FbFP single mutants are more stable than the WT. Most of the stabilizing mutants form a better core packing. The best triple mutant N107Y-N124Y-M111F increases the 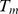 by 31°C without compromising the fluorescence quantum yield. The experimental test is by no means extensive but the effectiveness of the protocol has been demonstrated. The combined experimental and computational method adopted in our work adds a new tool to enhance protein thermostability, which can be extremely useful when conventional methods (e.g. directed evolution) fail.

## Supporting Information

Figure S1 Distribution of the thermostable mutations in the 3D X-ray FbFP structure (pdb code: 2PR5). The 40 mutants are: V23I/M/W/K, V25I, T30L/M, A33Y/L, V40I, T50M/I, T54L/Y, V75I/M, A81M, V88L/Y, V90I, N94M/Y, L106M, N107M/F, D109E, T117L, V120I/L/M, G121M/Q, N124I/M/F, Q129M, S139M and T141M. Residues highlighted in yellow are those with ΔΔG more negative than −1 kcal/mol. The stability was predicted by free energy calculations.
(DOCX)

Figure S2 A). Thermodynamic cycle built for free energy calculations. B). The intermediate state I designed to connect the X and Y mutants.
(DOCX)

Figure S3 Fluorescence emission spectra of FbFP at different temperatures. The excitation wavelength was set at 450 nm.
(DOCX)

Figure S4 Correlation time τ of ΔH (the Hamiltonian difference between two neighboring windows) as a function of λ in the F111 electrostatic (panel A) and vdw (panel B) transformation. The average correlation time is 3.0 ps for the electrostatic transformation and 2.5 ps for the vdw transformation.
(DOCX)

Table S1Δ*H*, 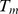, Δ*C_p_*, *α_F_*, *α_U_* values derived from the fluorescence curve fitting for FbFP WT and mutants.(DOCX)

Table S2 Contacts of selected residue side chains in the WT and mutants.
(DOCX)

Table S3 Hydrogen bond lists of selected residues.
(DOCX)

Table S4 Salt bridges formed with K22 in H22K mutant.
(DOCX)

Table S5 Frequency of a specific amino acid occurrence at selective sites of the WT and mutants from the alignment of 83 FbFP sequences.
(DOCX)
