# Supplementary figures and images for: Engineering a More Thermostable Blue Light Photo Receptor Bacillus subtilis YtvA LOV Domain by a Computer Aided Rational Design Method
Source: PLoS Comput Biol. 2013 Jul 4;9(7):e1003129. doi: 10.1371/journal.pcbi.1003129 (PMC3701716; doi:10.1371/journal.pcbi.1003129)

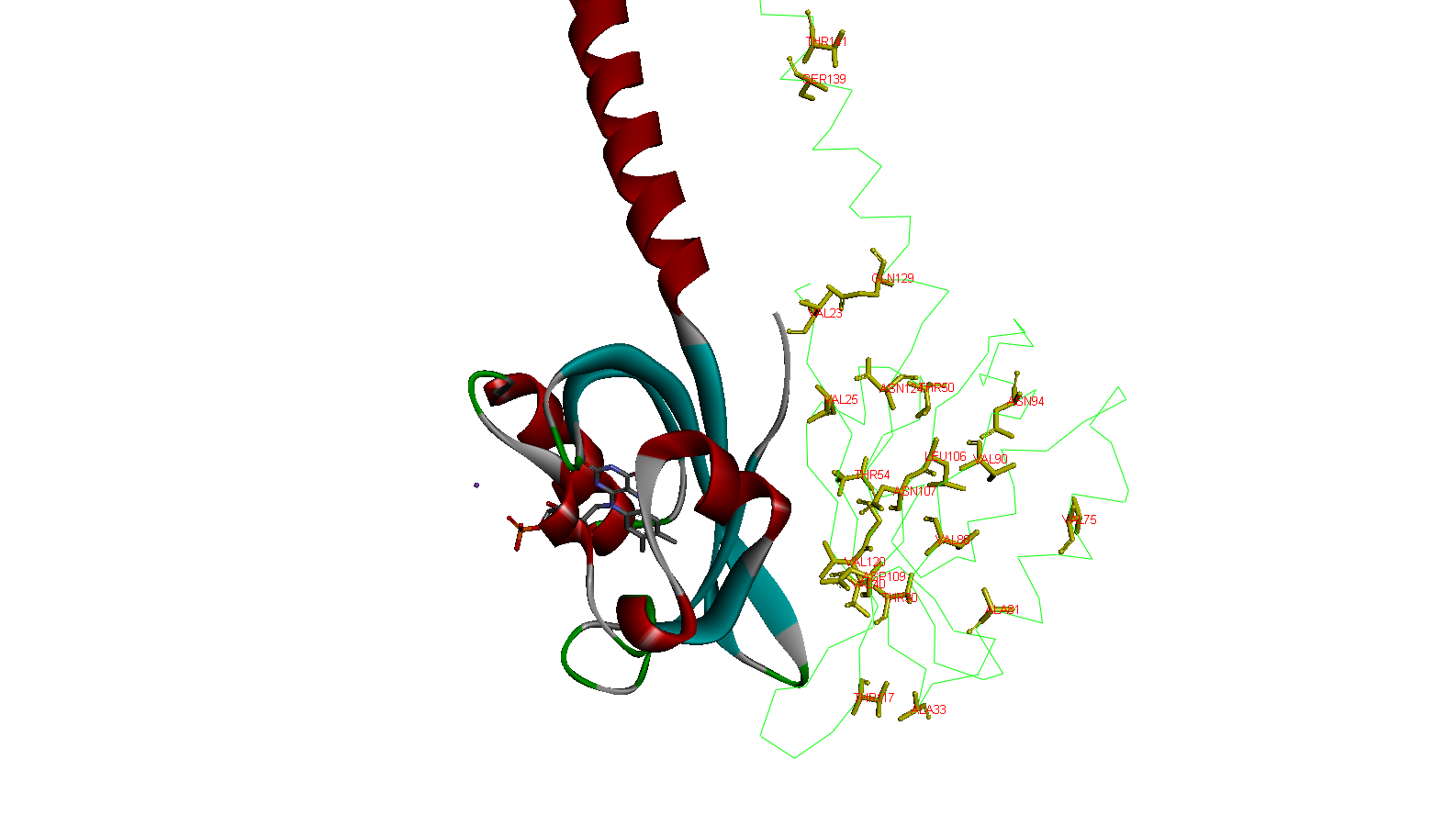

Supplement: Figure S1 — Distribution of the thermostable mutations in the 3D X-ray FbFP structure (pdb code: 2PR5). The 40 mutants are: V23I/M/W/K, V25I, T30L/M, A33Y/L, V40I, T50M/I, T54L/Y, V75I/M, A81M, V88L/Y, V90I, N94M/Y, L106M, N107M/F, D109E, T117L, V120I/L/M, G121M/Q, N124I/M/F, Q129M, S139M and T141M. Residues highlighted in yellow are those with ΔΔG more negative than −1 kcal/mol. The stability was predicted by free energy calculations. (DOCX) [file pcbi.1003129.s001.docx]

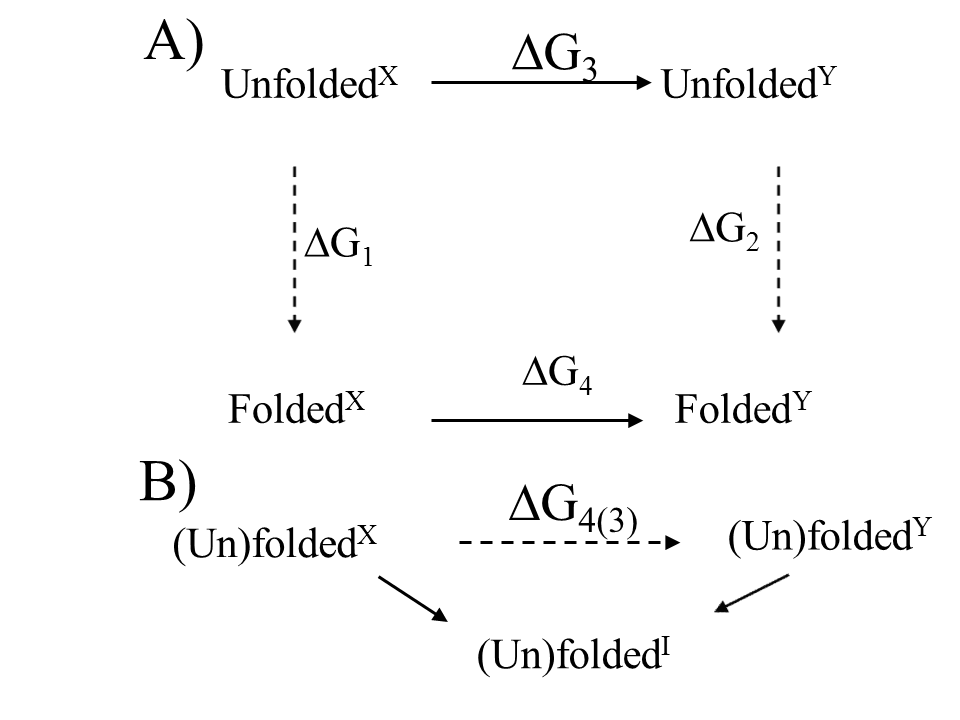

Supplement: Figure S2 — A). Thermodynamic cycle built for free energy calculations. B). The intermediate state I designed to connect the X and Y mutants. (DOCX) [file pcbi.1003129.s002.docx]

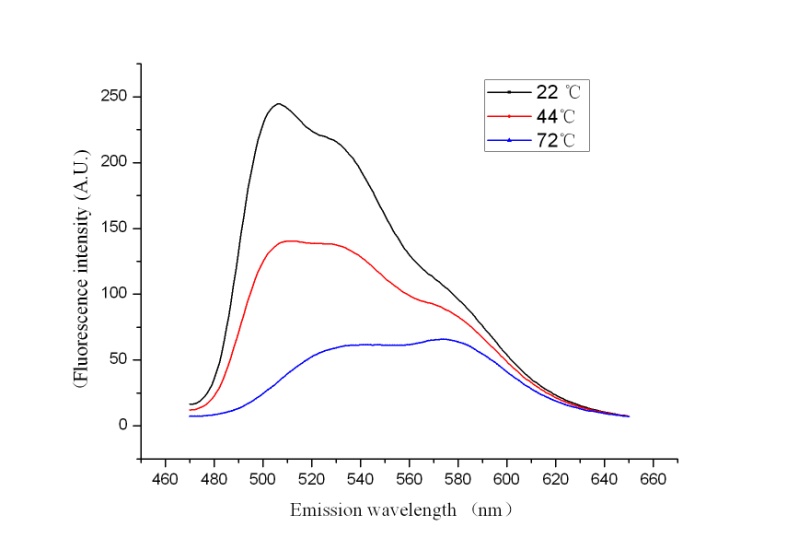

Supplement: Figure S3 — Fluorescence emission spectra of FbFP at different temperatures. The excitation wavelength was set at 450 nm. (DOCX) [file pcbi.1003129.s003.docx]

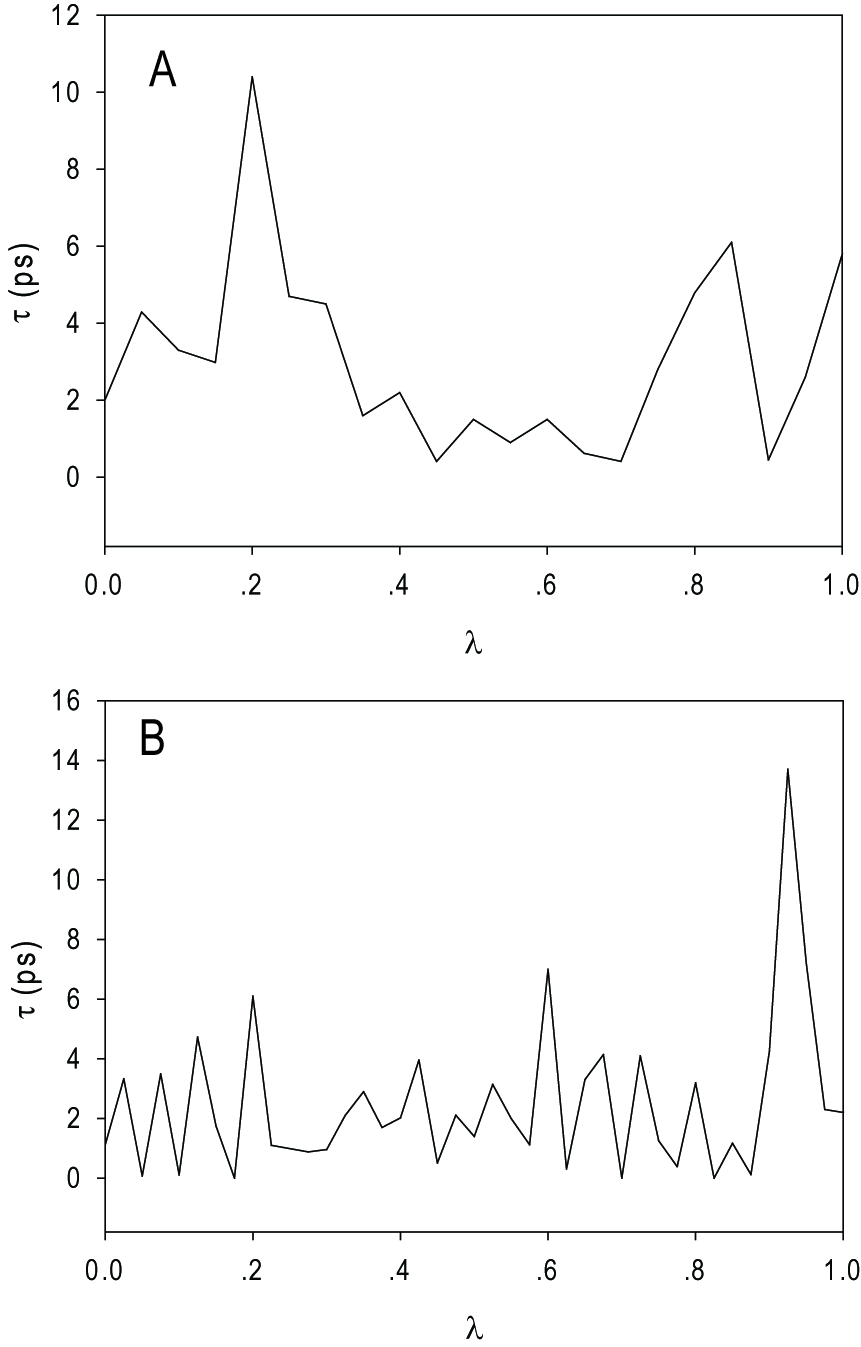

Supplement: Figure S4 — Correlation time τ of ΔH (the Hamiltonian difference between two neighboring windows) as a function of λ in the F111 electrostatic (panel A) and vdw (panel B) transformation. The average correlation time is 3.0 ps for the electrostatic transformation and 2.5 ps for the vdw transformation. (DOCX) [file pcbi.1003129.s004.docx]
