# Supplementary material for: Engineering a More Thermostable Blue Light Photo Receptor Bacillus subtilis YtvA LOV Domain by a Computer Aided Rational Design Method
Source: PLoS Comput Biol. 2013 Jul 4;9(7):e1003129. doi: 10.1371/journal.pcbi.1003129 (PMC3701716; doi:10.1371/journal.pcbi.1003129)
Supplement: Table S1 — ΔH, , ΔCp, αF, αU values derived from the fluorescence curve fitting for FbFP WT and mutants. (DOCX) [file pcbi.1003129.s005.docx]

|  | Δ*H*(kcal/mol) | Δ*C*_p_(kcal/mol·K) | *T*_m_(°C) | α_F_ | α_U_ |
| --- | --- | --- | --- | --- | --- |
| WT | 35.9±1.3^a^ | 0.89±0.06 | 42.8±0.3 | 109.8±1.8 | 14.1±0.3 |
| H22K | 33.8±1.0 | 0.67±0.03 | 45.3±0.2 | 107.4±0.9 | 18.2±0.4 |
| H22W | 61.7±2.1 | 1.54±0.1 | 49.4±0.2 | 102.0±0.8 | 18.6±0.4 |
| V25I | 32.3±1.7 | 0.66±0.05 | 46.6±0.5 | 120.7±2.2 | 17.7±0.7 |
| T30M  A33Y  T50M  T54Y  A81M  V88L  V90I  N107F  N107Y  D109E  M111F  V120I  N124F  N124Y  N107F_N124F  N107Y_V120I  N107Y_N124Y  N107Y_N124Y_H22W  N107Y_N124Y_M111F | 38.9±1.7  46.1±1.8  21.7±2.9  65.2±1.3  42.7±8.7  31.2±6.3  22.8±1.6  39.5±1.4  31.1±1.8  22.2±1.6  66.2±7.1  35.8±3.2  50.6±1.0  55.9±0.4  29.2±2.4  39.0±3.5  26.8±0.2  79.0±3.2  49.2±1.1 | 0.83±0.06  1.13±0.08  0.87±0.02  1.59±0.14  0.03±0.61  1.01±0.63  0.44±0.04  0.87±0.05  0.57±0.05  -0.26±0.17  -0.21±1.08  0.66±0.17  1.15±0.04  1.13±0.03  0.45±0.06  0.70±0.09  0.34±0.01  1.69±0.10  0.93±0.04 | 47.3±0.4  46.6±0.3  36.4±1.7  43.7±0.1  39.8±2.3  41.0±2.5  33.6±1.1  50.9±0.3  52.0±0.5  46.8±2.0  56.5±1.0  54.2±0.9  50.2±0.1  63.8±0.1  59.3±0.8  56.4±0.6  69.5±0.1  67.5±0.2  74.9±0.2 | 104.9±1.4  104.6±1.1  135.6±7.6  100.7±0.6  108.5±14.0  123.0±10.8  122.3±4.2  106.7±1.3  109.6±1.9  121.3±3.5  96.1±0.9  106.0±4.0  101.0±0.4  103.4±0.2  108.1±2.5  101.4±1.6  103.4±0.3  98.8±0.4  103.2±0.7 | 13.8±0.9  15.9±0.6  13.5±0.3  14.1±0.2  12.5±0.4  17.8±5.8  18.5±0.4  16.3±0.5  13.7±1.1  7.6±6.5  15.7±7.6  18.8±1.6  12.2±0.3  17.8±0.1  8.1±3.6  24.5±1.5  5.2±0.2  24.5±0.6  14.2±0.5 |

^a^*^.^* Errors were derived from 100 synthetic data with the random noise.
