# Supplementary material for: Engineering a More Thermostable Blue Light Photo Receptor Bacillus subtilis YtvA LOV Domain by a Computer Aided Rational Design Method
Source: PLoS Comput Biol. 2013 Jul 4;9(7):e1003129. doi: 10.1371/journal.pcbi.1003129 (PMC3701716; doi:10.1371/journal.pcbi.1003129)
Supplement: Table S2 — Contacts of selected residue side chains in the WT and mutants. (DOCX) [file pcbi.1003129.s006.docx]

|  | Subunit 1 | Percentage (%)^a^ | Subunit 2 | Percentage (%) |
| --- | --- | --- | --- | --- |
| H22 (WT) | E105 | 99 | Q129′ | 45 |
|  |  |  | Y132 | 30 |
|  |  |  | E133′ | 46 |
| K22(H22K) |  |  | E105′ | 31 |
|  |  |  |  |  |
| W22(H22W) | E105′ | 72 | Q44 | 43 |
|  | N124′ | 58 |  |  |
|  | D125′ | 96 |  |  |
|  | I126′ | 56 |  |  |
| N107(WT) | R24′ | 52 | V88 | 61 |
|  | T89 | 86 | T89 | 94 |
|  | I122 | 95 | I122 | 93 |
| F107(N107F) | R24′ | 73 | Q44′ | 76 |
|  | T89 | 88 | V88 | 61 |
|  | I122 | 79 | T89 | 76 |
|  |  |  | I122 | 33 |
| Y107(N107Y) | Y41′ | 92 | R24′ | 51 |
|  | N43′ | 88 | P87 | 85 |
|  | Q44′ | 97 | V88 | 82 |
|  | T89 | 89 | T89 | 89 |
|  | I122 | 96 | I122 | 48 |
| M111(WT) | Y41′ | 81 | Y41′ | 55 |
|  | Y118 | 83 | Y118 | 39 |
|  | I29′ | 50 | V120 | 36 |
| F111(M111F) | I29′ | 47 | I29′ | 53 |
|  | I29 | 52 | V40′ | 44 |
|  | Y41′ | 90 | Y41′ | 71 |
|  | Y118 | 92 | Y118 | 92 |
|  | V120 | 61 | V120 | 61 |
| V120(WT) | Y41′ | 87 | I29 | 53 |
|  | D109 | 98 | Y41′ | 63 |
|  |  |  | D109 | 87 |
|  |  |  | M111 | 36 |
| I120(V120I) | V27′ | 47 | V27 | 34 |
|  | I29′ | 46 | I29′ | 32 |
|  | I29 | 38 | I29 | 35 |
|  | Y41′ | 76 | D109 | 94 |
|  | D109 | 49 | M111 | 53 |
|  | M111 | 39 |  |  |
| N124(WT) | R24′ | 50 | V23′ | 47 |
|  | V25 | 72 | V25 | 71 |
|  |  |  | E105 | 43 |
| F124(N124F) | D21 | 90 | R24′ | 34 |
|  | D21′ | 83 | V25 | 43 |
|  | V23′ | 60 | E105 | 30 |
|  | V25 | 59 | F124′ | 66 |
|  | V25′ | 41 | Q129 | 69 |
|  | E105 | 44 |  |  |
|  | F124′ | 66 |  |  |
|  | I126 | 73 |  |  |
| Y124(N124Y) | V23′ | 76 | D21′ | 57 |
|  | R24 | 49 | H22′ | 41 |
|  | R24 ′ | 67 | V23′ | 71 |
|  | V25 | 33 | V25 | 78 |
|  | V25 ′ | 67 | V25′ | 56 |
|  | E105 | 94 | E105 | 94 |
|  |  |  | Q129 | 59 |

^a.^ Percentage is defined as the number of snapshots with the distance between side chain heavy atoms of the target residue and nonhydrogen atoms of the rest of the protein less than 4.0 Å divided by the total number of snapshots. Different percentages exist for different atom pairs from the two residues. For the sake of simplicity, only the largest percentage is listed in the table, demonstrating the close contact between the two residues. A cutoff of 30% is utilized, so that the residues with contact less than 30% are not included. Contacts from sequential residues are excluded. The analysis was based on 0.5 ns molecular dynamics simulations.
