# Supplementary material for: Engineering a More Thermostable Blue Light Photo Receptor Bacillus subtilis YtvA LOV Domain by a Computer Aided Rational Design Method
Source: PLoS Comput Biol. 2013 Jul 4;9(7):e1003129. doi: 10.1371/journal.pcbi.1003129 (PMC3701716; doi:10.1371/journal.pcbi.1003129)
Supplement: Table S3 — Hydrogen bond lists of selected residues. (DOCX) [file pcbi.1003129.s007.docx]

|  | Subunit 1 | Percentage (%)^a^ | Subunit 2 | Percentage (%) |
| --- | --- | --- | --- | --- |
| D109 (WT) | OH(Y41′)…OD1(D109)^b^ | 98 | OH(Y41′)…OD1(D109) | 57 |
|  |  |  | OH(Y41′)…OD2(D109) | 41 |
| E109(D109E) | ND2(N107)…OE1(E109) | 21 | OH(Y41′)…OE1(E109) | 86 |
|  | OH(Y41′)…OE1(E109) | 53 | OH(Y41′)…OE2(E109) | 15 |
|  | OH(Y41′)…OE2(E109) | 41 | ND2(N107)…OE1(E109) | 7 |
|  |  |  | ND2(N107)…OE2(E109) | 15 |
| H22(WT) | NE2(H22)…OE1(E105′) | 55 | NE2(H22)…OE1(E133′) | 29 |
|  | NE2(H22)…OE2(E105′) | 57 | NE2(H22)…OE2(E133′) | 27 |
|  | NE(R24)…ND1(H22) | 29 |  |  |
| Y107(N107Y) | OH(Y107)…OD1(D109) | 14 | OH(Y107)…OD1(D109) | 39 |
|  | OH(Y107)…OH(Y41′) | 10 | OH(Y107)…OD2(D109) | 18 |
|  |  |  | NH(Q44)…OH(Y107) | 89 |
| Y124(N124Y) | OH(Y124)…O(V23′) | 5 | OH(Y124)…O(V23′) | 27 |
|  | NH(V25′)…OH(Y124) | 53 | NH(V23′)…OH(Y124) | 16 |
|  |  |  | NE2(Q129)…OH(Y124) | 17 |

^a.^ Percentage is defined as the number of snapshots with the heavy distance less than 3.5Å and donor-hydrogen-acceptor angle larger than 150° divided by the total number of snapshots. Hydrogen bonds with percentage less than 5% are excluded. ^b.^ The hydrogen bond is written as donor heavy atom (donor residue) –acceptor heavy atom (acceptor residue). Prime denotes residues from the neighboring subunit.
