# Supplementary material for: Engineering a More Thermostable Blue Light Photo Receptor Bacillus subtilis YtvA LOV Domain by a Computer Aided Rational Design Method
Source: PLoS Comput Biol. 2013 Jul 4;9(7):e1003129. doi: 10.1371/journal.pcbi.1003129 (PMC3701716; doi:10.1371/journal.pcbi.1003129)
Supplement: Table S4 — Salt bridges formed with K22 in H22K mutant. (DOCX) [file pcbi.1003129.s008.docx]

|  | Subunit 1 | Percentage (%)^a^ | Subunit 2 | Percentage (%) |
| --- | --- | --- | --- | --- |
| K22 (H22K) | OE1(E105′) | 44 | OE1(E105′) | 40 |
|  | OE2(E105′) | 54 | OE2(E105′) | 45 |
|  |  |  | OE1(E133) | 35 |
|  |  |  | OE2(E133) | 43 |

^a.^ Percentage is defined as the number of snapshots with the heavy distance less than 5Å divided by the total number of snapshots.
