# Supplementary material for: Engineering a More Thermostable Blue Light Photo Receptor Bacillus subtilis YtvA LOV Domain by a Computer Aided Rational Design Method
Source: PLoS Comput Biol. 2013 Jul 4;9(7):e1003129. doi: 10.1371/journal.pcbi.1003129 (PMC3701716; doi:10.1371/journal.pcbi.1003129)
Supplement: Table S5 — Frequency of a specific amino acid occurrence at selective sites of the WT and mutants from the alignment of 83 FbFP sequences. (DOCX) [file pcbi.1003129.s009.docx]

| site | WT (%^a^) | Mutant (%^a^) | Top 3 high frequency mutants |
| --- | --- | --- | --- |
| 22 | H(20.5) | K(1.2) | E(33.7), H(20.5), L(15.7) |
|  |  | W(0) |  |
| 25 | V(56.6) | I(25.3) | V(56.6), I(25.3), A(4.8) |
| 30 | T(81.9) | M(0) | T(81.9), S(13.3), A(4.8) |
| 33 | A(7.2) | Y(0) | S(41.0), E(21.7), D(13.3) |
| 54 | T(3.6) | Y(4.8) | R(24.1), K(19.3), E(12.0) |
| 107 | N(19.3) | F(2.4) | T(38.6), N(19.3), R(19.3) |
|  |  | Y(1.2) |  |
| 109 | D(28.9) | E(18.1) | A(39.8), D(28.9), E(18.1) |
| 111 | M(6.0) | F(12.0) | L(31.3), I(31.3), V(27.7) |
| 120 | V(78.3) | I(15.7) | V(78.3), I (15.7), L(4.8) |
| 124 | N(15.7) | F(0) | K(45.8),N(15.7), D(14.5) |
|  |  | Y(2.4) |  |

^a^. The percentage at a site (the number in the parenthesis) is defined as the ratio of a specific amino acid occurrence divided by 83, which is the total number of sequences.
